# *NOTCH2NLC* mutation-positive neuronal intranuclear inclusion disease with retinal dystrophy: A case report and literature review

**DOI:** 10.1097/MD.0000000000033789

**Published:** 2023-05-12

**Authors:** Takayuki Katayama, Kae Takahashi, Osamu Yahara, Jun Sawada, Ken-ichi Ishida, Asuka Asanome, Hisako Endo, Tsukasa Saito, Naoyuki Hasebe, Mari Kishibe, Harumi Kanno, Satoshi Ishiko, Jun Sone

**Affiliations:** a Department of Neurology, Asahikawa City Hospital, Japan; b Division of Neurology, First Department of Internal Medicine, Asahikawa Medical University, Japan; c Department of Dermatology, Asahikawa Medical University, Japan; d Department of Ophthalmology, Asahikawa City Hospital, Japan; e Department of Ophthalmology, Asahikawa Medical University, Japan; f Institute for Medical Science of Aging, Aichi Medical University, Japan.

**Keywords:** case report, magnetic resonance imaging, neuronal intranuclear inclusion disease, *NOTCH2NLC*, retinal dystrophy

## Abstract

Neuronal intranuclear inclusion disease (NIID) is a neurodegenerative disorder that produces a broad spectrum of clinical conditions such as dementia, upper motor neuron involvement, extrapyramidal symptoms, and neuropathy. Some studies have reported ophthalmological conditions associated with the disease; however, the details of these conditions remain unclear.

**Patient concerns:**

We report a 63-year-old Japanese female with cognitive decline, blurred vision, photophobia, and color blindness at 52 years of age who was diagnosed with cone dystrophy. She also had anxiety, insomnia, depression, delusions, hallucinations, a wide-based gait with short steps, and urinary incontinence.

**Diagnoses, interventions, and outcomes:**

Magnetic resonance imaging revealed diffuse cerebral white matter changes and subcortical hyperintensity on diffusion-weighted imaging. Skin biopsy showed p62-positive intranuclear inclusions in sweat glands. *NOTCH2NLC* gene analysis revealed abnormal GGC expansion; therefore, NIID was diagnosed.

**Conclusion:**

*NOTCH2NLC* mutation-positive NIID may be associated with retinal dystrophy. Brain magnetic resonance imaging and skin biopsy are helpful diagnostic clues, and gene analysis is crucial for accurate diagnosis and appropriate management.

## 1. Introduction

Neuronal intranuclear inclusion disease (NIID) is a neurodegenerative disorder that produces a broad spectrum of clinical conditions such as dementia, upper motor neuron involvement, extrapyramidal symptoms, and neuropathy.^[1,2]^ It shows characteristic neuroimaging findings such as diffuse cerebral white matter changes and abnormal subcortical signals on diffusion-weighted imaging. Recent studies have revealed diagnostic skin biopsy findings, that is, intranuclear inclusions in sweat glands,^[3]^ and demonstrated that *NOTCH2NLC* gene analysis is crucial for diagnosing the condition.^[4]^ Some studies have mentioned ophthalmological conditions associated with the disease^[2]^ but the details of these conditions remain unclear.

Herein, we report a rare case of NIID with retinal dystrophy and a literature review.

## 2. Case report

A 63-year-old Japanese female was referred to our clinic with cognitive decline. She had been well until she developed blurred vision, photophobia, and abnormalities of color perception at the age of 52 years and was diagnosed with cone dystrophy. By the age of 61 years, she also had anxiety, insomnia, depression, jealous delusions, suicidal wishes, visual hallucinations, and urinary incontinence. She became forgetful at the age of 63. She had a history of bronchial asthma, hypertension, and dyslipidemia, which were treated continuously with medications. She had a history of smoking and alcohol drinking between the ages of 20 and 25 but had ceased these habits. The patient’s family history was unremarkable.

**Figure 1.**
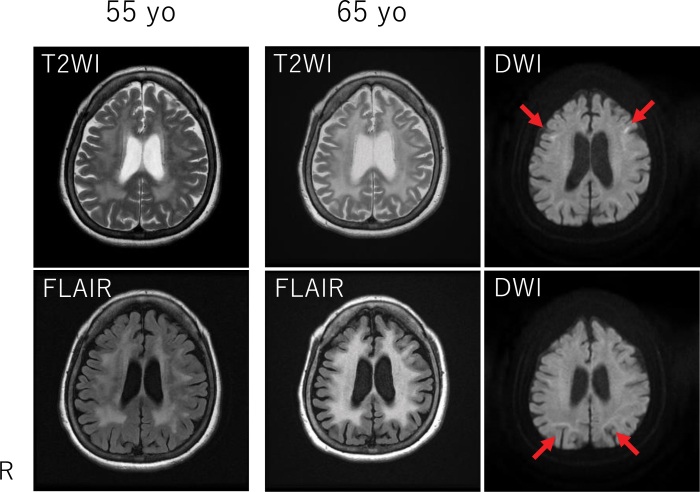
Brain magnetic resonance imaging scans of the patient obtained at the ages of 52 and 65 T2-weighted imaging (T2WI) and fluid-attenuated inversion recovery (FLAIR) showed progressive diffuse confluent white matter changes. Diffusion-weighted imaging (DWI) shows subcortical hyperintense signals in the frontal and parietal lobes (arrows).

**Figure 2.**
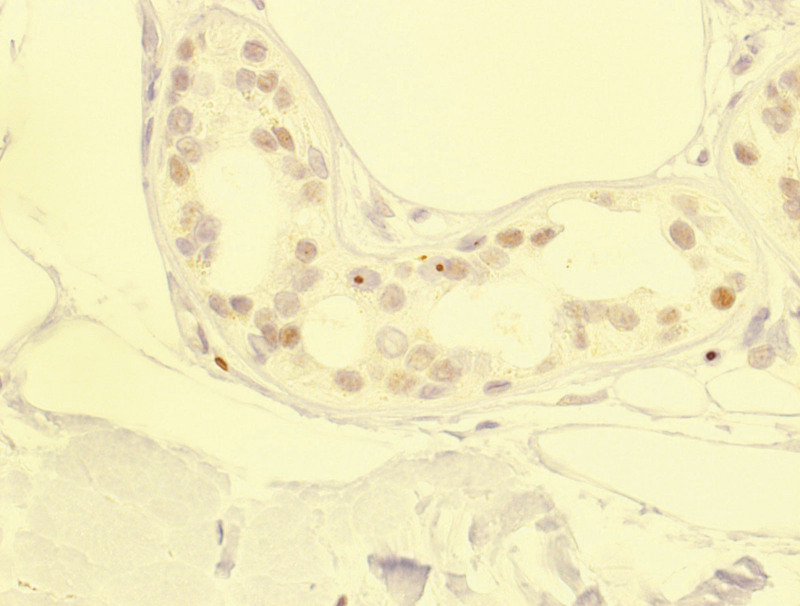
Skin biopsy. Immunostaining with anti-p62-antibody revealed intranuclear inclusions in the eccrine sweat glands (arrows). Bar = 20 μm.

**Figure 3.**
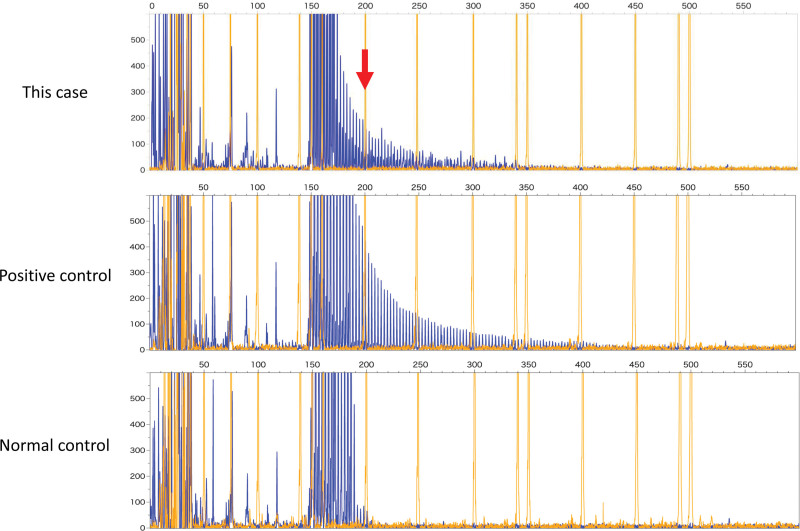
*NOTCH2NLC* gene analysis. A repeat primed polymerase chain reaction (PCR) test revealed serrated declining peaks in a >200-bp region (arrow), which suggested the presence of repeat expansion. bp = base pairs.

The patient’s physical examination findings were unremarkable. However, neurological examinations revealed dementia (Mini-Mental State Examination,12/30; frontal assessment battery, 6/18; Montreal Cognitive Assessment, Japanese version, 9/30) and decreased visual acuity. The patient experienced gait difficulties (wide, short steps). Brain magnetic resonance imaging (MRI) showed diffuse hyperintensity in the cerebral white matter on T2-weighted imaging and fluid-attenuated inversion recovery imaging and subcortical hyperintensities in the frontal and parietal lobes on diffusion-weighted imaging (Fig. 1). Brain perfusion scintigraphy with n-isopropyl-(^123^I)-p-iodoamphetamine revealed unremarkable changes (data not shown). A nerve conduction study demonstrated a mild reduction in tibial nerve velocity. A skin biopsy sample from the right forearm showed p62-positive intranuclear inclusions in sweat glands, together with fibroblasts and adipose cells (Fig. 2). Genetic analysis revealed abnormal GGC expansion and fluorescence amplicon length analysis confirmed the presence of 67/2 repeats (normal: ≤30) in the *NOTCH2NLC* gene (Fig. 3). Conversely, genetic analysis of fragile X mental retardation 1 did not detect any abnormal expansions (data not shown).

### 2.1. Ophthalmological examinations

**Figure 4.**
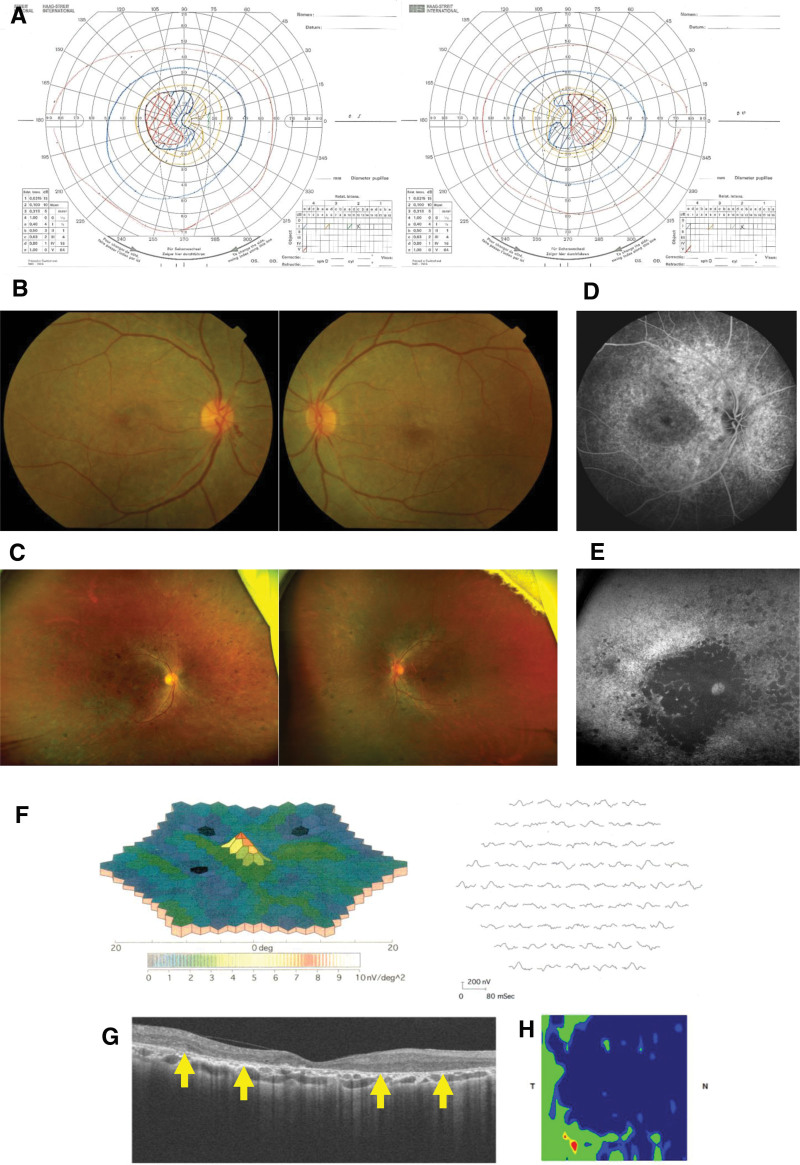
Ophthalmological findings. (A) Goldmann perimetry performed at the age of 53. Central scotomas were observed in both eyes. (B) Fundus findings obtained at the age of 53 (angle of view: 50°). Macular degeneration, arterial stenosis, and arteriosclerosis were seen. Arterial looping was also observed in the lower nasal area of the right eye. (C) Fundus findings obtained at the age of 65 (angle of view: 200°). Degeneration and pigmentation were seen throughout the retina. (D) Fluorescein angiography (FA) of the right eye performed at the age of 65. Hypofluorescence in the macular region was surrounded by hyperfluorescence. (E) Fundus autofluorescence (FAF) test of the right eye performed at the age of 65 (angle of view: 200°). Hypofluorescence was observed throughout the whole retina (especially the macula). (F) Multifocal electroretinography (ERG) of the right eye performed at the age of 53 Decreased sensitivity of the posterior pole was seen. (G) Optical coherence tomography (OCT) of the right eye performed at the age of 65 (horizontal image). Total loss of the ellipsoid zone (arrows) and irregular thinning of the retina were observed. (H) OCT color map image of the retinal thickness of the right eye (macular area: 5 mm × 5 mm). Green areas indicate normal thickness and blue areas indicate abnormal thinning; that is, thickness values that were smaller than 95% of those found in a database of normal thickness values with a Gaussian distribution (N: nasal; T: temporal).

At the age of 53 years, her decimal best-corrected visual acuity was 0.2 in the right eye (−0.5 diopters) and 0.2 in the left eye (+1.0 dpt), respectively. A color perception examination involving the panel D-15 test, standard pseudoisochromatic plates, and Ishihara test revealed total color blindness. Her light reflex was prompt. Goldmann perimeter test showed a central scotoma and a relatively narrow peripheral visual field (Fig. 4A). Fundoscopic examination revealed retinal degeneration mainly at the posterior pole at the age of 53 (Fig. 4B), followed by whole retinal degeneration and pigmentation at the age of 65 (Fig. 4C). Fluorescein angiography revealed hypofluorescence in the macular area surrounded by hyperfluorescence (Fig. 4D). Fundus autofluorescence test performed at the age of 65 years showed hypofluorescence throughout the retina, especially at the posterior pole (Fig. 4E). Flash and multifocal electroretinography were performed at the age of 53. Only multifocal electroretinography showed a decreased response at the posterior pole (Fig. 4F). Optical coherence tomography conducted at the age of 65 years showed total loss of the ellipsoid zone and irregular thinning of the retina, which was compatible with retinal dystrophy (Fig. 4G and H).

The patient received regular follow-up but her visual field and acuity deteriorated progressively (hand motion: 50 cm in both eyes at 65 years of age).

Written informed consent was obtained from the patient and her legal guardian/next of kin.

## 3. Discussion

We obtained detailed long-term data regarding the neuroimaging and ophthalmological findings of a case of NIID with retinopathy in which we detected progressive changes in the cerebral white matter and retina. To the best of our knowledge, only 15 cases of *NOTCH2NLC*-mutated NIID with retinopathy have been reported to date (Tables 1 and 2).^[5–9]^ These cases involve blurred vision, night blindness, color deficiencies, photophobia, and various neurological symptoms. It should be noted that some cases did not involve visual complaints, although ophthalmological tests have detected various abnormalities.^[7]^ Some cases of NIID involve progressive dementia; therefore, it is possible that such patients do not realize that they have visual symptoms. A recent review stated that 51.0% of patients with NIID have blurred vision,^[10]^ but the clinical and pathological features of NIID remain unclear.

**Table 1:** Summary of the clinical features and neuroimaging, skin biopsy, and genetic findings of cases of neuronal intranuclear inclusion disease with retinopathy.

No.	Author (reporting year)	Age/sex	Family history	Neurological symptoms	MRI lesions	Skin biopsy abnormalities	FMR1 mutations	NOTCH2NLC mutations	References
1	Omoto (2018), Hayashi (2020)	49F	+	Muscle weakness of extremities, atrophy, areflexia	+	+ (Ub)	?	Probable 94/15 GGC repeats	^[5,6]^
2	Nakamura (2020)	75M	−	None	?	?	?	330 bp (110 repeats)*	^[7]^
3		66F	−	None	?	?	?	400 bp (133 repeats)*	
4		73F	+	Reduced visual acuity	?	?	?	520 bp (173 repeats)*	
5		79F	+ (Aunt of No. 6)	Night blindness	?	?	?	350 bp (167 repeats)*	
6		75M	+	Night blindness	?	?	?	340 bp (113 repeats)*	
7		73F	+	Reduced visual acuity	?	?	?	370 bp (123 repeats)*	
8		81M	−	Reduced visual acuity, Night blindness, photophobia	?	?	?	~500 bp (167 repeats)*	
9	Hsia (2021)	68F	−	Cognitive impairment, unsteady gait	+	+	?	abnormal expansion	^[8]^
10	Liu (2021)	56M	+	?	+	? †	?	97 repeats	^[9]^
11		66M	−	?	+	? †	?	97 repeats	
12		29M	+	?	−	? †	?	>180 repeats	
13		65F	−	?	+	? †	?	94 repeats	
14		66M	+	?	+	? †	?	89 repeats	
15		69M	−	?	+	? †	?	84 repeats	
16	This case (2022)	63F	Unknown	Blurred vision, urinary incontinence, dementia, anxiety, insomnia, depression, jealous delusions, hallucinations, gait disturbance	+	+ (p62)	Normal	97/2 repeats	

bp = base pairs, F = female, FMR1 = fragile X mental retardation 1, M = male, Ub = ubiquitin, ? = not described, MRI = magnetic resonance imaging.

* These mutations were originally described in terms of repeat length (bp), which is convertible to repeat units (in parentheses), as described in the reference.

† These cases were diagnosed by genetic analysis and skin biopsies (the presence of ubiquitin-positive intranuclear inclusions); however, detailed pathological findings were not described.

**Table 2:** Summary of the ophthalmological findings of cases of neuronal intranuclear inclusion disease with retinopathy.

No.	Visual acuity (right/left)	Color perception	Visual field	Fundus lesions	ERG	OCT	FA	FAF
1	(0.2)/(0.9)	?	Constricted visual fields, relative central scotoma	Retinal degeneration with macular atrophy	Abnormal	Total disruption of the ellipsoid zone layer and thinning of the outer nuclear layer in both foveal areas	?	Hypo-autofluorescence in both posterior poles
2	(0.6)/(0.9)	?	Concentric constriction (Goldmann)	+	Abnormal	Abnormal	?	Abnormal
3	(1.2)/(1.2)	?	?	+	Abnormal	abnormal	?	Abnormal
4	(0.5)/(0.7)	?	?	+	NE	abnormal	?	Abnormal
5	(0.2)/(0.2)	?	Concentric constriction (Goldmann)	+	Abnormal	NE	?	Abnormal
6	(1.0)/(0.8)	?	Concentric constriction (Goldmann)	+	Abnormal	Abnormal	?	Abnormal
7	(0.3)/(0.15)	?	?	+	Abnormal	Abnormal	?	Abnormal
8	(0.5)/(0.4)	?	Concentric constriction (Goldmann)	+	Abnormal	Abnormal	?	Abnormal
9	(0.8)/(0.8)	Diffuse color discrimination error	Bitemporal visual field defects (Humphrey)	Diffuse peripapillary retinal pigment epithelium changes	Abnormal	Abnormal	?	Hypo-autofluorescence
10	(0.2)/(0.05)	?	?	None	Abnormal	Abnormal	?	Chorioretinal atrophy
11	(CF/40 cm)/(CF/40 cm)	?	?	Retinal pigmentation, macular hole	Abnormal	Abnormal	?	Chorioretinal atrophy
12	(0.8)/(0.8)	?	Normal	None	?	Normal	?	−
13	(1.0)/(0.9)	?	?	None	?	Abnormal	?	−
14	(0.2)/(0.3)	?	?	Retinal pigmentation	?	Abnormal	?	Chorioretinal atrophy
15	(0.25)/(0.6)	?	?	Macular degeneration	?	Abnormal	?	−
16	(0.2)/(0.2)	Total color deficiency	Central scotoma (Goldmann)	Macular degeneration followed by degeneration of the whole retina	Abnormal (multifocal ERG)	Loss of the ellipsoid zone and irregular thinning of the retina	Abnormal	Abnormal

The subject numbers in the left-hand column are identical to those listed in Table 1.

Visual acuity is expressed as decimal best-corrected visual acuity.

CF = counting fingers, ERG = electroretinography, FA = fluorescein angiography, FAF = fundus autofluorescence test, NE = not examined, OCT = optical coherence tomography, ? = not described.

However, little is known about the pathogenesis of retinal involvement in patients with NIID. However, NIID exhibits broad nervous system involvement; therefore, it is plausible that NIID-related retinopathy shares the same mechanism and involves the central and peripheral nervous systems.

Some neurodegenerative disorders, such as triplet repeat diseases, show repeat length-dependent severity,^[11]^ but it is uncertain whether a larger GGC repeat expansion in the *NOTCH2NLC* gene causes more severe clinical manifestations in NIID with retinopathy. Further studies are needed to determine the involvement of other genetic or acquired risk factors (e.g., smoking) in the acceleration of retinal degeneration in NIID.

A recent study revealed that NIID is genetically heterogeneous; repeat expansions in the *NOTCH2NLC* gene were found to be associated with NIID in patients of Japanese descent, but only a single European case harboring a pathogenic repeat expansion with a distinct haplotype structure was identified in a study of 11 European NIID cases based on whole-genome sequencing data from 20,536 participants in the 100,000 Genomes Project.^[10]^ Other studies have reported cases of NIID with retinopathy in Taiwan and China that involved repeated expansion of the *NOTCH2NLC* gene.^[8,9]^ It should also be noted that fragile X mental retardation 1 mutations produce similar MRI abnormalities and intranuclear inclusions in skin biopsy samples^[12]^ and therefore, accurate diagnosis and classification based on genetic analysis are required.

The histopathological features of retinopathy in NIID cases with *NOTCH2NLC* mutations remain unknown. Electrophysiological studies have shown rod-cone dysfunction in most cases; thus, the suggestion of Nakamura et al^[7]^ that photoreceptors are the primary sites of NIID defects seems reasonable. In contrast, Hsia et al^[8]^ described a case involving damaged ganglion cells and photoreceptors. Some variations or inconsistencies in the clinical features of NIID could be partly explained by differences in the clinical stage because NIID is a progressive disorder.

Ophthalmological abnormalities observed in NIID seem to have received insufficient attention, and it is important to perform appropriate evaluations of visual function in patients with NIID. NIID is thought to be a rare disorder; however, the number of reported cases has been growing in recent years because of diagnostic advances in MRI, skin biopsies, and genetic tests. Early and accurate diagnosis, better genetic counseling, and more innovative treatments are required.

## Acknowledgments

We thank the patient and her relatives for agreeing to the publication of this report. We also thank Professor Hidetoshi Takei (Department of Pathology, Asahikawa Medical University Hospital, Japan) and the staff of the Department of Pathology at Hokkaido University for performing pathological examinations.

## Author contributions

**Conceptualization:** Takayuki Katayama.

**Data curation:** Kae Takahashi, Jun Sawada, Ken-ichi Ishida, Asuka Asanome, Hisako Endo, Tsukasa Saito, Mari Kishibe, Harumi Kanno, Satoshi Ishiko, Jun Sone.

**Project administration:** Takayuki Katayama, Osamu Yahara.

**Supervision:** Osamu Yahara, Naoyuki Hasebe, Satoshi Ishiko.

**Writing – original draft:** Takayuki Katayama.

**Writing – review & editing:** Osamu Yahara, Naoyuki Hasebe, Satoshi Ishiko.
